# The Influence of Metabolic Factors and Diet on Fertility

**DOI:** 10.3390/nu15051180

**Published:** 2023-02-27

**Authors:** Klaudia Łakoma, Olha Kukharuk, Daniel Śliż

**Affiliations:** 1 3rd Department of Internal Medicine and Cardiology, Medical University of Warsaw, 02-091 Warsaw, Poland; 2 Polish Society of Lifestyle Medicine, 00-388 Warsaw, Poland; 3 Medical Center of Postgraduate Education, Public Health School, 01-813 Warsaw, Poland

**Keywords:** fertility, fertility diet, nutrition, plant-based, pro-fertile diet, nutritional patterns, reproductive health, infertility, metabolic factors

## Abstract

Infertility is a disease globally affecting 20–30% of the reproductive age female population. However, in up to 50% on recorded cases, problems with infertility are ascribed to men; therefore, it is important to popularize healthy eating also in this group. During the last decade, it has been observed that society’s lifestyle changed drastically: reduced energy expenditure in physical activity per day, increased consumption of hypercaloric and high-glycemic-index foods with high content of trans fats, and reduced consumption of dietary fiber, which negatively affects fertility. Increasing evidence points to a link between diet and fertility. It is becoming clear that well-planned nutrition can also contribute to the effectiveness of ART. The low-GI plant-based diet appears to have a positive effect, especially when it is based on Mediterranean dietary patterns: rich in antioxidants, vegetable protein, fiber, MUFA fatty acids, omega-3, vitamins, and minerals. Importantly, this diet has been shown to protect against chronic diseases associated with oxidative stress, which also translates into pregnancy success. As lifestyle and nutrition seem to be important factors affecting fertility, it is worth expanding knowledge in this regard among couples trying to conceive a child.

## 1. Introduction

Infertility is a disease concerning both, male and female reproductive system. It can be defined as the failure in achieving clinical pregnancy in the period of 12 or more months with the assumption of regular unprotected sexual intercourse [1,2,3,4].

It is estimated that 15% of reproductive-age couples in the world experience difficulty getting pregnant—at least 48.5 million couples [5,6,7]. This global health problem affects 20–30% of the female population of reproductive age in modern society [8]. In contrast, only about 7% of all men worldwide suffer from male infertility [9]. Among 20% of couples unsuccessfully trying to get pregnant, the problem of infertility affects both women and men [10]. Estimated that female infertility accounts for only 35% of all infertility cases, whereas about 20–30% of infertility cases are solely due to males, but generally male factors are identified as the main cause in 50% of infertility problems overall [1,11]. The remaining 15% of couples unable to conceive have “unexplained infertility” [12].

Infertility can be overwhelming for couples diagnosed with this medical problem, they are more likely to experience stress and have a higher risk of developing serious mental disorders, such as anxiety and depression, compared to healthy couples [13,14]. Taking these data into account, all possible solutions should be sought. In recent years, researchers have been looking for lifestyle factors that favor fertility. One of them is a properly balanced diet. Nutrition can have a negative or positive effect on the fertility of both women and men and the impact depends on both quantitative and qualitative properties of the diet, such as the caloric content of each macronutrient (carbohydrates, fat, and protein), as well as the specific acid profiles fatty acids, proteins, and carbohydrates. All components of nutrition constitute a dietary pattern [11,13,15,16,17].

Based on current knowledge, it can be confirmed that the Mediterranean dietary (MD) pattern is remarkably positive for fertility. A growing interest, noticed in the scientific evidence, suggests that increased consumption of plant-based protein, rich in antioxidants, fiber, and low-glucose-index (GI) carbohydrates have an equally important effect on fertility. This aspect indicates that the plant-based nutritional model can lead to a positive effect on couples’ fertility [15,16,18,19,20].

The number of people around the world following plant-based diets has grown enormously in the last few years. In the general population likewise in the scientific community of Western countries, the interest for this particular nutritional model has increased remarkably and became the one of the major eating patterns [21]. A survey published in 2019 it turned out that 40% of consumers are trying to reduce their animal-protein intake, whereas 10% avoid red meat altogether [22].

Many studies have shown the positive effects of a well-planned plant-based diet on health. This dietary pattern has been linked to many favorable health effects: reduced risk of diabetes and cardiovascular disease, potential beneficial effect on body weight and BMI in overweight people [21,23,24], and lower risk of depression [25]. 

Continuing the topic of a plant-based diet, it is worth adding that public-health agencies recommend this model of nutrition, especially high-quality plant foods (such as fruits, vegetables, whole grains, and nuts) to help prevent chronic illness [26].

According to our knowledge, in the current literature it is hard to find the connection between particular factors like insulin resistance, oxidative stress, healthy aging, weight reduction, low-glycemic-index plant-based diet, and infertility. Therefore, it is reasonable to determine the efficacy of plant-based diets in promoting reproductive health in order to supply adequate nutritional advice to clinical practice. 

## 2. Body Weight and Infertility

Many studies have looked at the effect of low body mass index (BMI) on reproductive ability. It has been shown that inadequate diets, whether low-calorie or unhealthy, excessive caloric intake, can disrupt the physiological reproductive function and greatly increase the risk of infertility [11,12,27]. NICE clinical guidelines also indicate body weight as an important factor in the context of reproduction [28]. According to the World Health Organization (WHO), underweight is classified as a BMI < 18.5. If a BMI is equal to or greater than 25 kg/m^2^, it is considered overweight, whereas a BMI higher than 30 kg/m^2^ defines obesity [29]. Underweight, overweight, and obesity have detrimental effects on several functions of the human body, including reproductive health [11,12,27].

Low BMI can be a negative factor of male and female infertility. As a result of fat loss and the resulting hormonal imbalance, there is an increased risk of fertility problems. In men, being underweight is often associated with malnutrition and an unhealthy lifestyle, which can lead to hormonal imbalance and reduced semen quality [30,31].

In the case of women with a BMI < 18.5, unfavorable pregnancy outcomes and problems with infertility have been reported. Women who are underweight (BMI < 19 kg/m^2^) have been shown to have four times longer time to get pregnant than women with correct weight [32]. Furthermore, in underweight women chronic energy deficiency can inhibit the hypothalamic–pituitary–gonadal (HPG) axis by affecting the gonadotropin-releasing hormone (GnRH) pulse generator. Inhibition of GnRH secretion leads to a cascade of inhibitory effects, including a reduction in gonadotropin secretion, a delay in follicular development, and an inhibition of gonadal steroid synthesis. Malnutrition caused by low food availability is not common in developed countries, but it can occur in eating disorders (ED) that affect women of childbearing age. Similar relationships are observed with relative energy deficiency in sport (RED-S); it includes menstrual disorders caused by low energy availability, which also results in anovulation [31,32].

However, the most common cause of female infertility is ovulatory dysfunction. It accounts for approximately 25% of infertility diagnosis, and 70% of women with anovulation have PCOS (polycystic ovary syndrome) [12]. Research has shown that abdominal obesity is a common symptom of PCOS. The effect of excess adipose tissue on the endocrine system is assessed as unfavorable. Moreover, dysfunction of this tissue can negatively affect follicle development. There is a strong link between insulin resistance, hyperinsulinemia, and infertility in PCOS patients. It is believed that insulin resistance leads to ovulation disorders and abnormal endometrial structure [33,34,35]. Importantly, women with PCOS are at a higher risk of developing type 2 diabetes, regardless of age and BMI. As obesity increases, the incidence of type 2 diabetes is increasing significantly; however, PCOS increases the relative risk in thin women [36]. Furthermore, given the current background of rising obesity rates among women of reproductive age, being overweight and obese prior to conception may be a barrier to pregnancy. Obesity clearly increases women’s risk of miscarriage, poor pregnancy outcomes, and impairment of fetal well-being [32,34]. Additionally, it is worth mentioning that women with excess body weight often suffer from irregular menstruation, ovulation disorders, endometrial pathology, and infertility [27]. Obese women were shown to have more than twice the risk of ovulatory-disorder infertility [37]. Analogously to obese women, some researchers showed that hormonal imbalance may be involved in reduced semen quality in men with high BMI [30].

Obesity interferes with reproductive potential, especially through changes in the HPG axis, impaired testicular steroidogenesis, and metabolic disorders including insulin, cytokines, and adipokines. Importantly, excess body fat has a negative impact on sperm parameters, including concentration, mobility, viability, and normal sperm morphology [38]. Metabolic disorders, such as obesity, type 2 diabetes, and insulin resistance are associated with the deterioration of fertility mainly as a result of the generation of oxidative stress, which is considered to be one of the main factors leading to reduced sperm quality and an increased risk of infertility, as well as hormonal disorders. Moreover, hyperglycemia has a negative impact on sperm motility and the fertilization process [39,40,41].

It is now known that up to 90% of male infertility causes are down to low sperm count, low sperm quality, or both [10]. Over the past 50 years, there has been an overall decrease in sperm concentration in men in Europe by 32.5% [42]. Supplementarily, the latest research data show that nutrition is one of the main factors directly related to sperm quality and that lifestyle is crucial in maintaining normal reproductive capacity [7,11,43,44].

Many researchers, recognizing that obesity is a potentially harmful factor for male reproduction, have focused their attention on examining the effects of weight loss on sperm quality through nutritional interventions. Some researchers emphasize that weight reduction should be used as a treatment for infertility in obese people [30,39]. In overweight women prior to conception, weight loss is an important factor in the success of conception by regulating the hormonal balance and finally inducing spontaneous ovulation [18].

It should be emphasized, however, that these results suggest that different types of infertility exist, require different approaches, and are not always aimed at weight loss. Low and high BMI and related metabolic implications are a risk factor for fertility. Understanding the most beneficial type and composition of diet is critical to the sustainable and effective management strategies that underpin healthy pregnancy and lifelong well-being in this clinical population [7,11,16,27,30,35,37,39,44].

## 3. Dietary Patterns and Infertility

As current studies indicate, dietary patterns may have a great influence on fertility-disorder development when perceived rather as a whole system instead of individual food constituents. This is according to the fact that people generally consume the composite meals and not separated nutrients [2,32,45,46,47]. Dietary patterns can be defined as the amount, proportion, variety and/or combination of different foods and drinks in the diets, as well as the daily/weekly frequency of consumption. Moreover, analysis of the effects of eating patterns may be more of a predictor of the relationship between diet and disease than a consideration focused on individual foods or nutrients [48,49]. It was shown that the lifestyle of fertile and infertile women differed especially in terms of nutrition and physical activity. Likewise, infertile and fertile men had significant differences in inappropriate health behavior and physical activity [50].

Many publications have compared the impact of particular nutritional models on fertility. The Western pattern has been shown to negatively affect fertility, and an inverse correlation has been observed in the context of the Mediterranean nutritional pattern [51,52,53]. The MD is characterized by high consumption of plant-derived food (vegetables, legumes, fruit, nuts, cereals, and seeds), fatty saltwater fish, low-fat dairy and poultry, and whole-grain cereal products with a low consumption of simple sugars (sweets) and red meat. Olive oil is the main source of fat and alcohol consumption should be moderate [32,46]. However, alcohol consumption is a leading risk factor for the global burden of disease and causes a significant health loss. The level of consumption that minimizes health loss has been shown to be zero. These results suggest that even a moderate amount has negative health effects [54].

Furthermore, the traditional Mediterranean diet is one of the healthiest dietary patterns and has been linked to many beneficial health effects: decreased risk of all-cause mortality, cardiovascular disease, cancer, and other chronic diseases [46,52]. In women of childbearing age, the MD seems to reduce the risk of weight gain and insulin resistance, which may increase the likelihood of pregnancy. This is due to the fact that insulin directly affects the function of the ovaries; therefore, insulin sensitivity and glucose metabolism may have a significant impact on ovulation and fertility in women. It is worth mentioning that hyperinsulinemia is strongly related with hyperandrogenism, which also exacerbates endocrine disorders in women and contributes to difficulties with conception. The risk of anovulatory infertility is correlated with oxidative stress. For this reason, antioxidants also seem to be important for proper ovulation [9,55,56,57,58,59,60]. Moreover, some antioxidants are positively related to sperm quality and thus may help to improve the quality and fertility of male sperm [61]. In fact, the MD pattern is rich in several nutrients that have been proven to have benefits in terms of sperm motility [52]. In a cross-sectional study of 225 men from couples attending a fertility clinic in Athens, men in the highest tertile of the MedDietScore were less likely to have normal sperm concentration, total sperm count, and sperm motility compared to men in the lowest tertile of the score [62]. It is suggested that the MD should be recommended for couples undergoing in vitro fertilization [32]. On the other hand, there is limited evidence of an association between nutritional patterns and in vitro fertilization outcomes [47]. However, what is interesting is that it has been shown that the Mediterranean model increased successful pregnancies by 40% among couples undergoing IVF (in vitro fertilization) [63]. Vegetables and fruits form the basis of healthy nutrition models that improve the quality and fertility of semen [7,43,52,64].

A completely different effect on ovulation and semen quality was noted in relation to Western-model nutrition, which negatively affects endocrine metabolisms [16,17]. Components in this dietary pattern that have a negative influence mostly include high-glycemic-index carbohydrates, sweets, sweetened beverages, large amounts of animal protein (especially red and processed meat), saturated fatty acids, and trans-fatty acids. It could also be described by a low consumption of fresh fruits and vegetables, dietary fiber, vitamins, unrefined grains, low-fat poultry, and sea fish [17,52,65]. 

In 2007, the data of the prospective Nurses Health Cohort Study II (NHS II) were published and the results were used to create a pattern of “fertility diet,” consisting of a lower intake of animal protein with a higher supply of vegetable protein, a high iron content, an increased supply of monounsaturated fatty acids with a simultaneous lower consumption of trans-fatty acids, consumption of high-fat dairy products, and the presence of carbohydrate products with a low glycemic index. The study was conducted in women and showed that participating in this nutritional pattern was associated with a lower risk of fertility impairment due to other factors [37].

Protein obtained from red meat and poultry has been shown to significantly increase the risk of infertility due to anovulation. Interestingly, no negative effects from fish and egg proteins were observed. Moreover, it has been shown that consuming 5% of energy from plant protein instead of animal protein reduces the risk of anovulatory infertility by over 50%. The difference may be due to the different influence of plant and animal proteins on insulin and insulin-like growth-factor I (IGF-I) secretion. The potentially beneficial effect of plant protein on fertility may arise from the fact that the insulin response is weaker with the consumption of plant protein than with animal protein. Furthermore, the exchange of carbohydrates with vegetable protein also has a positive effect. Substituting 5% of the energy requirements of carbohydrates with vegetable protein can reduce the risk of ovulation disorders by up to 43% [20]. Taking the above-mentioned facts into consideration, it can be confirmed that the consumption of plant protein has a positive influence on fertility. 

It is worth underlining that plant-based diets have significant potential in preventing or treating several serious chronic diseases, including obesity, type 2 diabetes, cardiovascular disease, and cancer, which has been described in many studies. Predominantly plant-based dietary patterns emphasizing a higher consumption of fruits, vegetables, legumes, whole grains, nuts, seeds, and vegetable oils, and including lower consumption of or exclusion of animal products have gained significant attention in recent years [24,66,67,68,69,70]. In addition, consumers are increasingly changing their diet and limiting their meat consumption. Moreover, the plant-based food retail market has grown faster than the general food retail market, and for health reasons, plant-based alternatives are expected to continue to grow in popularity [48].

Data from three prospective cohorts were prospectively followed [24], with 76,530 women in the Nurses’ Health Study (NHS) (1986–2012), 81,569 women in NHS II (1991–2017), and 34,468 men in the Health Professionals Follow-up Study (1986–2016), and it was found that healthy groups of plant foods such as whole grains, fruits, vegetables, tea, and coffee were factors significantly contributing to the lower risk of type 2 diabetes. In addition, plant-based diets have been shown to promote weight control, improve the intestinal microbiome, and be anti-inflammatory [23,24,71].

The anti-inflammatory effects of plant-based diets have been well proven [72,73,74]. Into the bargain, it has been shown that many natural plant-derived nutrients can positively affect mitochondria by modulating their metabolism, biogenesis, and redox status. Protecting mitochondrial function with these compounds may be important in explaining their beneficial effects on male reproductive performance [15]. Consumption of animal foods has been shown to negatively affect sperm motility, and an inverse correlation is seen in vegan groups that consume only plant-based foods [75].

Considering these facts, a diet based on plants with a low glycemic index has great potential for reproductive health for both men and women. However, its components and sources of individual micro- and macroelements are important because they determine the influence of the diet on fertility [16,17,75]. Fast food can also be vegan, but as is well known, its effect on reproduction is negative. The quality of plant-based diets is also important [16]. On the one hand, in the case of fertility disorders in people with excess body weight, a plant-based diet may promote weight loss [23,76]. On the other hand, in the case of low body weight, a poorly planned iron-poor diet may negatively affect ovulation in women [77].

Therefore, it is important to determine exactly which components of plant-based diets have a positive effect on fertility. 

## 4. The Role of Oxidative Stress and Insulin Resistance in Fertility

### 4.1. The Impact of Oxidative Stress

Many scientific studies have shown that oxidative stress has a negative impact on the fertility of both women and men [78,79,80,81,82].

It has been shown that the system’s defense against reactive oxygen species (ROS) can be overloaded if the production of ROS is maintained at a high level, which may cause disease conditions. Oxidative stress (OS) has been found to play a key role in the pathogenesis of infertility in both men and women. It is characterized by an imbalance between pro-oxidative molecules, including reactive oxygen and nitrogen species, and the antioxidant defense [81,83,84].

The occurrence of OS is caused by the overproduction of ROS. ROS play an important role not only as secondary messengers in many cascades of intracellular signaling, but interestingly, they also influence pathological processes concerning the female genital organs, and in men they affect sperm production. Imbalances between pro-oxidants and antioxidants could lead to a number of female and male reproductive diseases [78,81,82,85,86]. OS is predominantly caused by many lifestyle-related factors, most of them modifiable. The modern lifestyle associated with processed food and lack of exercise plays an important role in oxidative-stress induction [87].

It has been shown that an unhealthy hypercaloric diet, excessive consumption of saturated and trans-fatty acids, a high glycemic index, and a low nutrient density can increase the oxidative stress that causes carbohydrate disturbance. Insulin resistance and diabetes mellitus are related to the deterioration of fertility in women and men, mainly due to the generation of high oxidative stress, which is the main cause leading to an increased risk of infertility and hormonal disorders [19,86,88].

The solution to the problems with excess free radicals Is antioxidants—substances that neutralize them. In fact, the term “antioxidant” refers to the chemical property of donating electrons. In some situations, some substances act as antioxidants, and in others they become pro-oxidants, which depend on the chemical composition of the environment in which they are currently located [87,89]. There are many types of antioxidants, and their roles in the body and how they work are different. It is a misconception that one antioxidant can be replaced by another to produce the same effect. In fact, everyone has their own unique biological properties. Scientific research on antioxidants shows that more is not necessarily better. It should be underlined that unhealthy eating habits and unbalanced lifestyle cannot be compensed by eating superfoods. The free radicals as well as antioxidants can have a beneficial effect on the body. Hence, the most important is the balance—aimed not at the negative role attributed to free radicals but concerned at the positive role of antioxidants. That is why a well-balanced diet is very important, as well as moderate physical activity [87].

It is worth noting that antioxidants are ingredients present in fruits and vegetables (FAVs). With increased consumption of FAVs, a lower risk of chronic disease is seen when the diet is predominantly plant-based. Fruits and vegetables are rich in bioactive substances and they are the reason why it is worth choosing a balanced diet based on various sources of vegetable consumption. Moreover, the health benefits of antioxidants often also result from other substances present in food—not necessarily a specific type of antioxidant, but the synergistic action of several substances. For this reason, a well-balanced diet is better than supplementation. Antioxidants from berries and green vegetables are of particular importance. In addition, since a typical plant-based diet includes a wide variety of antioxidant-rich foods, it is likely that consuming these foods will strengthen your antioxidant system [16,78,87,90,91,92,93]. Importantly, a plant-based diet has been shown to protect against chronic diseases associated with oxidative stress [92,94,95,96].

Oligozoospermia, which is characterized by low number and quality of sperm, is responsible for 90% of male infertility. Nevertheless, the results of the studies showed that not all men who show normal parameters in a routine semen analysis are fertile. It is worth noting that the hidden factor was OS, which is now recognized as an important and probable cause of idiopathic male infertility [78,97].

Superoxide dismutase (SOD) is one of the most significant antioxidant enzymes and is involved in the conversion of anionic superoxide (O_2_^−^) into hydrogen peroxide (H_2_O_2_). The reaction is an important and necessary step in the antioxidant pathway during both normal cellular metabolism and various pathological processes [98]. It was suggested that changes in SOD status are related to the risk of developing disorders as well as their course and exacerbation. It has been shown that changes in the expression level, concentration, and activity of SOD are related with the occurrence of cancer, cardiovascular, metabolic, and neurodegenerative diseases [99,100]. Moreover, sperm oxidative stress is associated with decreased implantation rates in in vitro fertilization [78].

SOD is believed to be involved in the development of oocytes and the ovulation process, since the activity of this enzyme has been found in developing follicles, graaf membranes, postovulatory follicles, and follicular fluid and ovaries [101].

Considering the substantial role of chronic low-grade inflammation in the pathogenesis of numerous chronic diseases, including infertility, there is a need to implement the strategic nutritional approach by expert dietitians so as to design appropriate anti-inflammatory dietary interventions [102].

It is worth noting that insulin resistance has been suggested to be a causative factor in increased oxidative stress in women with PCOS. Insulin resistance causes hyperglycemia, which triggers the release of reactive oxygen species from mononuclear cells and causes further oxidative stress [79]. Moreover, circulating markers of oxidative stress in women with PCOS have been shown to be abnormal regardless of the weight [80]. It has been shown that postprandial hyperglycemia caused by the supply of large amounts of high-glycemic-index carbohydrates is associated with the intensification of inflammation and oxidative stress through the production of reactive oxygen species, which is why a well-planned diet is so important [102].

### 4.2. The Role of Insulin Resistance

Insulin resistance is identified as an impaired biological response to insulin stimulation of target tissues, mainly liver, muscle, and adipose tissue. Insulin resistance interferes with glucose removal, causing a compensatory increase in beta-cell insulin production and hyperinsulinemia. The consequence may be further metabolic complications, including hyperglycemia, hypertension, dyslipidemia, visceral obesity, and increased markers of inflammation [103].

It is indicated that glucose metabolism and insulin sensitivity can have a significant effect on ovulation and fertility in women. Insulin has a direct influence on the function of the ovaries [16]. It is suggested that insulin resistance may lead to ovulation disorders and abnormal endometrial structures. It has been shown that there is a strong association between insulin resistance, hyperinsulinemia, and infertility in PCOS patients [35]. The clinical pregnancy rate and the miscarriage rate has been shown to be higher when the male partner is diabetic [104]. Current research results suggest that obesity and diabetes negatively affect sperm parameters in men and are associated with low testosterone levels, which translates into fertility problems [105]. As in the case of obesity, studies have shown a correlation between an increase in diabetes incidence and a decrease in fertility rates [106]. Hyperglycemia has a negative impact on sperm motility and the fertilization process [11,39].

Moreover, studies have shown that 59% of men with diabetes have erectile dysfunction. Hyperglycemia leads to an increase in the level of ROS, an increase in advanced glycation end products (AGEs), an inhibition of endothelial nitric-oxide synthase metabolism, and a decrease in endothelial synthesis and the release of nitric oxide, leading to erectile dysfunction [107]. What is interesting is that it has been shown that as the number of metabolic-syndrome factors increases, the risk of erectile dysfunction increases. A relationship between metabolic syndrome and erectile dysfunction in men from infertile couples has also been demonstrated [108,109]. The major changes in semen quality induced by diabetes mellitus include a reduction in sperm density and motility, and an increase in sperm DNA fragmentation, and apoptosis [110]. In addition to the negative effects on sperm count, motility, and DNA integrity, lower ejaculate volumes were also observed in diabetic men [111].

It has been shown that the high susceptibility of sperm to oxidative damage is correlated with poor conception, pregnancy loss, congenital defects, poor embryo development and childhood cancer [78]. 

### 4.3. Antioxidants from Supplements and Diet

Men with impaired fertility have been shown to have lower levels of antioxidants in their semen compared to fertile men [82]. In such a situation, dietary supplementation with antioxidants may improve the concentration and motility of sperm, including the influence on DNA fragmentation and even the pregnancy rate [112]. Several studies in the literature have shown that increased consumption of fruit and vegetables, which are rich in antioxidants, is associated with a higher percentage of motile sperm in fertile and infertile men. Vegetables and fruits form the basis of healthy nutrition models that improve the quality and fertility of semen [75,113,114]. At the moment, there are no well-planned studies on a group of men assessing the effect of antioxidant supplementation on positive pregnancy outcomes. However, based on seven randomized studies, it is suggested that such supplementation in infertile men may positively affect fertility [64,115,116].

In the context of male fertility, zinc is a very important diet component because an adequate level of it in semen is essential for proper steroidogenesis and testicular development, as well as sperm production, maintenance of normal function, morphology, and cell count. Ultimately, it is necessary for the proper course of fertilization [117,118,119,120]. Which results from its strong antioxidant effect; an adequate amount of zinc in the semen plasma shows a protective effect [117,121]. On the other hand, it was shown that supplementation with folic acid and zinc by men seeking treatment with their partner in an infertility clinic did not contribute to a significant improvement in semen quality compared to placebo. These data do not confirm the validity of supplementation of these ingredients by male partners in the treatment of infertility [122]. 

Selenium also seems to be important. Several studies have found lower levels of selenium in the semen of infertile men compared to the healthy population. It is worth mentioning that both selenium deficiency and excess may result in abnormal sperm parameters and fertility disorders. Selenium increases sperm motility and vitality, which increases the chances of pregnancy, but also protects sperm DNA against oxidative stress [43,119,123,124,125]. In addition, vitamin C and tocopherol have a strong antioxidant effect, so it is very important that the diet be rich in FAVs [126]. Lycopene, a powerful antioxidant that reduces DNA damage due to oxidative stress but also increases sperm count and survival, seems to have a promising positive effect [127].

In the context of a vegan diet, reduced consumption of animal proteins can result in a deficiency of vitamin B12, zinc, calcium, and selenium, which can negatively affect reproductive health [119,125,128]. It has also been shown that plant-based diets lack vitamin D, iodine, and iron. For this reason, supplementation may be mandatory in some cases [129,130].

The aforementioned points provide solid evidence of the negative effects of insulin resistance and oxidative stress on a couple’s fertility. It is worth noting here that better adherence to a general and healthy plant-based diet was associated with a lower risk of type 2 diabetes, the opposite effect is observed with lower adherence. Interestingly, in both men and women, better adherence to a general and healthy plant-based diet for four years was correlated with a lower risk of type 2 diabetes in the following four years. Higher consumption of healthy plant food groups (especially whole grains, fruits, vegetables, tea and coffee) is correlated with lower risk of type 2 diabetes through various biological pathways such as weight management, anti-inflammatory effects, antioxidants and improvement of the gut microbiome through higher intake fiber and polyphenols, and lower saturated fat intake [24]. Additionally, plant-based diets that are high in fiber and polyphenols are also associated with a variety of gut microbiota that produce metabolites that have anti-inflammatory functions that can help manage disease processes, which can prove very beneficial in the context of infertility in couples [131].

## 5. Carbohydrates and a Diet with a Low Glycemic Index and Load

When describing the fertility-promoting nutrition pattern, attention should be paid to both insulin sensitivity and glucose metabolism, as both can significantly affect the fertility of men and women [32,132,133].

When discussing the topic of carbohydrates, the glycemic index and load are of particular importance [16]. It has been shown that eating foods with a high glycemic index and meals with a high glycemic load may lead to metabolic complications and increase the risk of insulin resistance, diabetes, dyslipidemia, and oxidative stress, which adversely affect fertility and reproductive function [32,109,133].

The mechanism by which they exert their negative effects is due to their effect on the sensitivity of tissues to insulin [11,16,39,55,88]. In the case of fertility problems in women, insulin affects ovarian function and ovulation by participating in the follicular response to gonadotropin. High glycemic index and load are correlated with higher fasting-glucose levels, hyperinsulinemia, and insulin resistance, which are also associated with higher concentrations of IGF-I and androgens. This naturally may lead to exacerbation of endocrine disorders in women and thus may be related to impaired oocyte development [16,55,133].

On the other hand, in the case of men, a diet with a high glycemic index may indirectly lead to fertility disorders, mainly due to the generation of oxidative stress, which is considered one of the main factors leading to reduced sperm quality and an increased risk of infertility. Hyperglycemia adversely affects sperm motility and may increase the risk of hormonal and immune disorders. In addition, obese men with insulin resistance and type 2 diabetes have been shown to be much more likely to experience hypogonadism [11,88,134]. 

A study was concerned on examination of 18,555 women which had no history of infertility. All of them were planning or became pregnant during the study and the results have shown that the women in the highest quintile of total carbohydrate consumption were at a 78% higher risk of anovulatory infertility. The authors of the study claim that the amount and quality of carbohydrates in the diet may be the crucial determinants of ovulation and fertility for women [19].

Continuing this topic, it was shown that a diet with a high glycemic index and low dietary-fiber content is strongly correlated with inflammation, which negatively affects the fertility of both sexes. Moreover, fructose is believed to have a strong proinflammatory effect [102]. It has also been observed that the consumption of sweetened, carbonated beverages negatively affects fertility, which may also reduce the chances of reproductive success with ART (Assisted Reproductive Technology) [135,136,137]. Moreover, consumption of sugar-sweetened drinks correlated with lower sperm motility in healthy young men. It has also been shown that higher consumption of fruit and vegetables is associated with improved sperm parameters [15,138]. In a prospective cohort study of 3628 women planning to become pregnant, women who reported consuming three or more daily soda drinks had a 52% lower pregnancy rate compared to women who reported no soda consumption. Interestingly, no association was found between coffee consumption and fertility [139]. In this context, these results suggest that sugar consumption has an adverse effect on female fertility. Moreover, there is evidence that high sugar consumption is associated with lower sperm quality and increased male infertility [44].

In the context of men, it is clearly visible that with the spread of the Western model of a diet rich in processed carbohydrates with a high glycemic index, the parameters assessing semen quality have deteriorated. This diet is associated with decreased male fertility [11,15].

In the review, which included seven studies, all included studies used a diet containing less than 45% of total energy derived from carbohydrates, but the approaches to nutritional interventions differed significantly. Nevertheless, it was shown that five of them had beneficial changes in female reproductive hormones. Four of the seven illustrated significant improvement in menstrual cycles and/or ovulation rates on a low-carbohydrate diet. Four out of seven saw significant improvements in fasting insulin and testosterone. Three out of seven showing improvement in pregnancy rates in the intervention group. The authors of the review suggested that in overweight women prior to conception, weight loss is an important factor in successful conception by regulating the hormone balance and finally inducing spontaneous ovulation [18].

Moreover, a meta-analysis and systematic review of 10 randomized trials showed that the use of low-glycemic-index diets was associated with decreased testosterone levels in women with PCOS, further demonstrating the positive effect of this diet on hormonal regulation [140].

However, the use of a very-low-calorie ketogenic diet is primarily associated with weight reduction, improved carbohydrate metabolism, and a significant decrease in insulin resistance and circulating markers of inflammation. Taking this into account, it may apply in the case of infertility. However, there is a lack of reliable research on long-term effects [141]. For women, the more they stick to a low-carbohydrate diet, the greater the chance of pregnancy and having regular periods. Additionally, this caloric restriction appeared to be more critical in alleviating hyperandrogenism. Low-carbohydrate diets have been shown to improve pregnancy rates, reduce the risk of miscarriage, and optimize ovulation function [35]. Interestingly, replacing carbohydrates with vegetable protein can have a positive effect. Replacing 5% of the energy requirement of carbohydrates with vegetable protein was associated with a reduction in the risk of ovulation disorders by as much as 43% [20]. 

Dietary fiber intake in excess of the recommended dose has been shown to be associated with an increased risk of anovulation. It has been suggested that this is due to lowered hormone concentrations as a result of high fiber intake, especially the water-soluble fraction. Each 5 g/d increase in total fiber intake was associated with a 1.78-fold increased risk of an anovulatory cycle [142]. It has also been found that infertile women consume too few high-fiber foods like FAVs [143]. Based on the conducted research, it is also necessary to emphasize the role of fiber in the context of male fertility; a proper supply along with diet is necessary to maintain proper reproductive function. The positive mechanism of action is probably also based on the binding of unconjugated estrogens, which is directly related to a lower level of estrogens in the plasma [7]. On another note, the positive effect on reproduction of a diet rich in fiber may be due to its effect on lowering blood glucose levels, which is associated with a low dietary glycemic load and index [133].

A popular factor associated with carbohydrate products is the topic of gluten’s effect on fertility. As recommended, excluding gluten from the diet in people without celiac disease is not beneficial. Moreover, gluten-free diets have been shown to have lower nutritional value compared to traditional diets [144,145]. This is a risky nutritional intervention for fertility, given that it involves eating less fiber and more saturated fatty acids and foods with a higher glycemic index [146].

Taking into account the above facts, in order for a plant-based diet to be pro-fertile, it should be balanced so that meals have a low glycemic load, are based on products with a low glycemic load, the amount of fiber is neither too low nor too high, and should be anti-inflammatory. It seems that limiting carbohydrate supply is also of key importance in the prevention of infertility. 

## 6. Plant and Animal Protein

Another important component of a fertility diet for couples is protein [15,16,20]. A low-protein diet has been identified as a potential risk factor for male infertility as it can lead to a significant reduction in the weight of the testicles, epididymis, and seminal vesicles, as well as a decrease in serum testosterone [15]. Conversely, reports on the influence of a high-protein diet in the literature are contradictory [147]. In addition, an increase in protein consumption may lead to a correct carbohydrate–insulin balance, which may be important in the treatment of anovulatory infertility in women [148].

Chavarro et al. [20] showed that the consumption of animal protein is associated with a higher risk of infertility due to anovulation. On the other hand, the consumption of plant-based protein increases the fertility of women > 32 years of age. The authors suggest that this may be due to the different effects of plant and animal proteins on insulin and IGF-I secretion. After consuming plant-based protein, the insulin response is weaker than after consuming protein. The insulin response is weaker with the consumption of vegetable protein than with animal protein. Interestingly, women who consumed more animal protein also consumed more saturated fatty acids than women who consumed little animal protein. Less activity was also observed in the group of women who consumed large amounts of protein. This factor may increase the correlation between ovulation disorders and animal-protein consumption. It is worth underlining that consuming 5% of energy from vegetable protein instead of animal protein reduced the risk of anovulatory infertility by over 50%. Interestingly, among the women of the NHS-II cohort, one additional serving of meat (chicken, turkey, red meat, fish, and processed meats) per day, while maintaining constant calories, was associated with a 32% increase in the risk of ovulatory infertility. The findings suggest that replacing animal-protein sources, particularly chicken and red meat, with vegetable-protein sources may reduce the risk of infertility due to anovulation [20].

It was shown that the consumption of protein, especially animal protein, negatively correlated with testosterone concentration in healthy women, which indicates that androgens play a very important role in the regulation of ovarian function and thus female fertility. Moreover, excessive androgen signaling appears to be a major factor in androgen-related reproductive disorders, as it negatively affects the pathways regulating ovarian-follicle dynamics. Interestingly, protein intake was not related to estradiol, progesterone, LH (luteinizing hormone), and FSH (follicle-stimulating hormone) levels. This study also showed that there is no correlation between the consumption of total, vegetable, and animal protein (without dairy protein) and the number of antral follicles in women experiencing infertility. On the other hand, high protein intake from dairy products resulted in a reduced number of antral follicles, which is a biomarker predicting the number of primary ovarian follicles [16,149,150,151].

In a study of 2217 women with anovulatory PCOS and with normal ovulation, women with normal ovulation had a significantly lower proportion of meat in their diet compared to women with ovulation disorders. Processed red meat has been shown to have a particularly negative effect on fertility [152]. It is worth noting that red-meat products have a particularly negative impact on health; its consumption is associated with numerous adverse health effects, which also affects fertility. Moreover, the consumption of plant protein at the level of 5% of energy requirement instead of carbohydrates was associated with the reduction of the risk of ovulation disorders by as much as 43% [20]. The potentially positive effect of plant protein on fertility may be related to the improvement of insulin sensitivity and lower postprandial secretion of this hormone compared to animal protein [16]. 

Moreover, Karayiannis et al. [62] found that low intake of meat and high consumption of fruits, vegetables, and whole grains are linked with increased total sperm count. In a case-control study of 30 men with reduced semen quality and 31 normozoospermic controls, it was reported that the control group that consumed more tomatoes, lettuce, and fruits had a significantly higher percentage of motile sperm compared to infertile cases that consumed more meat and yogurt [153]. One study analyzed cases of infertility with poor semen quality and found that increased consumption of meat and processed foods correlates with poor semen quality [154]. As far as we know, the first available study comparing sperm parameters between vegans and non-vegans showed that the plant-based diet had a positive and beneficial effect on male fertility. Vegan groups had a significantly higher percentage of rapid progressive sperm as well as a higher percentage of motile sperm [75]. Interestingly, it was observed that among infertile couples, total meat consumption by men was also positively related to their partner’s total meat consumption and the Western eating pattern [155]. The same study showed that poultry consumption was positively associated with the fertilization index, whereas consumption of processed meat was negatively associated with the fertilization index among couples who underwent conventional in vitro fertilization. Furthermore, this had no effect on clinical pregnancies or live birth rates [155].

On the other hand, fish-consumption studies suggest that the fertility benefits of eating fish may outweigh the harm caused by some of the environmental contaminants of these foods. The relationship between protein-rich food intake and ART outcomes was also assessed in a prospective cohort study. Researchers estimated the effect of substituting one source of dietary protein for another on the probability of live birth. Consuming fish instead of any other protein-rich food (i.e., all other meats, eggs, legumes, soy, and nuts) was consistently related to greater odds of live birth. The contrast was greatest when fish was consumed instead of processed meats [156]. One source of protein in the plant-based diet is soy, whose possible negative effects on the endocrine system, in particular through isoflavones (IS), has raised concerns among some researchers, especially given previous animal studies [2]. However, evidence from human studies (although it has its limitations) has shown that soy consumption does not help or harm couples trying to get pregnant naturally, and interestingly, in the ART context it has been shown that isoflavone consumption can increase the chance of a successful pregnancy [2,157,158,159]. Moreover, logically, soybeans can contain many other phytochemicals, e.g., phytosterols, phytic acid, flavonoids, etc., so the activity of soy cannot be related to the activity of its isoflavones alone. Supplementarily, even at high concentrations, they did not show any pronounced effect on fertility. However, this may be due to the little research done on the subject [160]. In the case of men, there was also no evidence of a negative effect of soy consumption on fertility and no effect on testosterone and sex-hormone-binding globulins [161,162]. Concerns remain that the phytoestrogens (isoflavones) in soy may cause male feminization. An extended meta-analysis indicated that irrespective of the dose and duration of the study, exposure to neither soy protein nor isoflavones was negatively affected by soy [163]. Moreover, the consumption of soy food and soy isoflavones was not related to sperm motility, sperm morphology, or ejaculate volume [164]. On the other hand, a cross-sectional study in women found that women who consumed higher amounts of soy isoflavones were 13% less likely to become pregnant compared to women who consumed standard doses [165].

Summing up, based on the research carried out, it can be speculated that a higher proportion of plant protein than animal protein is more favorable in terms of fertility. 

## 7. Fats

Fatty acids are another dietary component that is being investigated for possible interference with reproductive mechanisms. The effect of individual fatty-acid fractions on fertility is different. The right amount and quality of fatty acids consumed is of great importance in the context of the prevention of fertility problems. Both excessive and insufficient dietary fat contribute to fertility disorders. Both appear to have negative effects on fertility [166]. However, in the case of ovulation disorders, the most important thing is the quality, not the quantity of fat [167].

Trans-fatty acids (TFAs) appear to have the most negative impact on fertility. TFAs have been found to negatively affect ovulation function in women, promoting insulin resistance, and their increased consumption leads to an increase in inflammatory markers [168,169,170]. TFAs have pro-inflammatory properties and, in addition to increasing insulin resistance, they also increase the risk of developing type 2 diabetes or other metabolic disorders, including PCOS, which may also adversely affect fertility [166,168,171]. Overall, most of the evidence supports the negative effects of diets high in trans-fatty acids and low in PUFAs (polyunsaturated fats) on reproductive performance in healthy women [172]. TFAs can also negatively affect semen quality and spermatogenesis. According to some sources, an excess of saturated fat, often associated with the Western pattern, also has a negative effect on fertility [7,154]. It has been reported that trans-fatty acids from fried/conventional baked or industrially processed food are inversely associated with the total sperm count. In addition, the content of trans-fatty acids in semen is associated with lower quality, as well as a lower concentration of sperm in the ejaculate [11]. The main sources of harmful fatty acids in the diet include fast food, ready-made confectionery, salty and sweet snacks, and processed and red meat [173]. A negative correlation was observed between the consumption of TFAs contained in sweets, hard margarines, and fast food and normal ovulation. Among women, the conversion of 2% of energy obtained from polyunsaturated fatty acids or monounsaturated fatty acids to TFAs was associated with a doubled risk of anovulatory infertility. The more TFAs in a woman’s diet, the greater the risk of ovulation disorders. Moreover, any increase in TFA energy by 2% instead of carbohydrate energy was also associated with anovulatory infertility [166].

Saturated fatty acids (SFAs) and TFAs have been found to have a particularly negative effect on ovulation [174]. It was also shown that women with PCOS consumed more animal fat and more saturated fat, which had a negative effect [143,175,176].

Saturated fat content, which may be particularly high in red meat, has been independently linked to lower sperm concentration in males [177]. PUFAs, conversely, have been shown to yield reproductive benefits in both men and women. A cross-sectional study of men showed that higher intake of omega-3 fatty acids was associated with significantly more favorable sperm morphology [177]. It was also shown that eating red meat before IVF had a negative effect on the development of the embryo and the likelihood of clinical pregnancy. An inverse correlation was observed with higher fish consumption [178]. It is worth adding that the consumption of oily marine fish by women has been shown to have a positive effect on fertility, regardless of the degree of exposure of the fish to contamination [179].

Avocados are an important part of the fertility diet, as they contain far greater amounts of the important nutrients folate and potassium, which are normally under-consumed in a mother’s diet. Although not part of a “traditional” Mediterranean-style diet, avocados meet the requirements for the antioxidant- and fiber-rich fruit category and have a fatty-acid profile that is naturally high in monounsaturated fats (MUFAs), which are linked to better pregnancy outcomes [180]. MUFAs are also important in the context of fertility, as they can bind to the PPAR-γ receptor, thus reducing inflammation and positively affecting fertility [181].

A meta-analysis of 16 randomized controlled trials showed a positive association between semen quality parameters and omega-3 supplementation in infertile men. Importantly, health-promoting dietary models that included fish and seafood were also associated with better semen quality in observational studies [182]. Women who had higher amounts of omega-6, linoleic acid and omega-3 in their diets were more likely to become pregnant than those who consumed less of these nutrients [183]. In addition, the consumption of omega-3 fatty acids and omega-3-rich foods by women can increase the likelihood of conception by reducing the risk of pregnancy loss [184]. PUFA supplementation has a positive effect on female fertility by influencing the concentration of LH and FSH, dominant-follicle maturation, oocyte quality, and the induction of ovulation [167]. It is worth mentioning that omega-3 fatty acids derived from oily marine fish were associated with increased levels of progesterone, whereas docosapentaenoic acid was correlated with decreased anovulation [185]. PUFA intake appears to have profitable effects on female reproduction, namely on oocyte quality and embryo implantation [186,187,188]. It has also been shown that high intake of TFAs and low intake of ω-3 fatty acids were associated with decreased fertility [172]. In addition, PUFAs are precursors for prostaglandins, which are important in aspects of reproductive physiology such as successful implantation, and have anti-inflammatory properties [188]. It is also possible that PUFAs contained in fish mediate intracellular signaling pathways in embryo implantation [189]. Nonetheless, omega-3 fatty-acid intake should still be recommended to these groups as a part of a healthy fertility diet [13].

The consumption of walnuts also has a positive effect on fertility due to the omega-3 content. Consuming 75 g of walnuts daily for 12 weeks was associated with longer sperm viability, motility, and morphology. Interestingly, according to another study, adding 60 g of a nut mix to the Western diet, in addition to improving the above-mentioned parameters, also resulted in an increase in the number of sperm [190,191].

A popular procedure among couples trying to conceive is elimination of various products such as dairy. A total of 232 women who underwent 353 in vitro fertilization (IVF) procedures were examined. Total dairy consumption was found to be positively associated with live birth in women ≥ 35 years of age under ART [192]. A cohort BioCycle study showed that a higher frequency of anovulation was noted in women consuming higher amounts of cream and yogurt, which suggests the potential importance of specific dairy-food intake on ovulatory function among healthy, regularly menstruating women [193]. On the other hand, high consumption of low-fat dairy products may increase the risk of anovulatory infertility, whereas consumption of high-fat dairy products may reduce this risk. In addition, lactose (the main carbohydrate in milk and dairy products) may not affect fertility within the normal range of human consumption. Adding one serving of whole milk without increasing energy expenditure reduces the risk of ovulatory infertility by more than 50%. This response was likely due to the fact that higher-fat dairy products had a higher estrogen content and produced a lower IGF-1 increase compared to lean dairy products [194]. On the other hand, dairy products have been shown to support female fertility because women who drank more than three glasses of milk a day had a 70% reduction in the risk of infertility compared to participants who drank no milk [195]. This was demonstrated in the context of men that low-fat milk and skim milk were positively associated with several parameters of semen quality [7]. Since they are low in saturated fat, choosing low-fat dairy products seems to be beneficial [43].

However, there is solid evidence to suggest that eating yogurt may protect against the development of type 2 diabetes, and for this reason it is worth including it in a pro-fertility diet [196]. In addition, consumption of fermented dairy products was associated with a lower risk of developing diabetes by affecting the gut microbiota, and thus tissue-insulin sensitivity and glucose tolerance [197,198].

Interestingly, the consumption of low-fat dairy products, particularly low-fat milk, was associated with a higher sperm concentration, whereas the consumption of cheese was associated with a lower sperm concentration, but only among former or current smokers. Moreover, fish consumption was correlated with total sperm count and morphology [199]. 

Overall, diets high in monounsaturated and polyunsaturated fatty acids appear to have a positive effect on fertility, unlike trans-fatty acids and an excess of saturated fatty acids in the diet. A plant-based diet should be low in processed foods, sweets, and fast food (Table 1). 

## 8. Conclusions

The literature on the relationship between diet and fertility is expanding rapidly. The evidence presented above suggests that various components of diet and lifestyle may contribute to reducing the risk of fertility problems in the general population of reproductive age, and may also be an effective treatment for women and men already experiencing infertility. Nutritional advice can be crucial in treating infertility as there is strong evidence that healthy eating habits adopted before conception in both men and women of childbearing age have a beneficial effect on fertility. What is more, well-planned nutrition can also translate into the effectiveness of ART. A well-balanced plant-based diet with a low glycemic index, with the lowest possible proportion of processed foods, seems to have a positive effect on fertility, especially if planned by a dietitian and not deficient in minerals or vitamins. However, due to the risk of deficiency, in some cases supplementation should be considered: vitamin B12, vitamin D, zinc, selenium, calcium, iodine, and iron.

It has been shown that eating a plant-based diet with a high intake of vegetable protein, antioxidants, fruits, vegetables, nuts, legumes, olive oil, and adequate fiber intake is associated with improved fertility. 

In contrast, unhealthy plant-based diets characterized by high consumption of sweetened beverages, sugar, junk food, refined grains, baked goods, and processed foods adversely affect fertility through potential mechanisms such as exacerbation of inflammation. For a plant-based diet to have a positive effect on fertility, it should follow a Mediterranean pattern and be anti-inflammatory. Moreover, it should not be deficient in protein, and both excess and deficiency of fiber may have a negative effect.

## Figures and Tables

**Table 1:** Overview of the literature on the relation between diet and fertility.

Diet Component/Dietary Pattern	Active Components/Set of Components	Impact on Fertility
Vegetables and fruits	Plant protein	Plant protein is a super-fertile ingredient. Consuming 5% of energy from plant protein instead of animal protein reduces the risk of anovulatory infertility by over 50% [20]. Consuming only plant products in a vegan diet has a positive effect on semen quality [75].
	Antioxidants, vitamins, minerals, folic acid	Antioxidants are important for proper ovulation [9,55,56,57,58,59,60] and quality of semen [7,43,52,61,64]. A high amount of antioxidants in the diet greatly increases fertility, especially the antioxidants from berries and green vegetables [16,78,87,90,91,92,93].
	Fiber	A diet rich in vegetables and fruits promotes fertility [15,16,78,87,90,91,92,93,138]. Adequate supply of fiber along with the diet is crucial; too low and too high a dose negatively affects fertility [7,133,142,143].
Refined grains, sweets, sweetened beverages, meals with a high glycemic load	Excess simple sugars, low content of fiber	Eating foods with a high glycemic index and meals with a high glycemic load may lead to metabolic complications and increase the risk of insulin resistance, diabetes, dyslipidemia, and oxidative stress, which adversely affect fertility and reproductive function [32,109,133]. The consumption of sweetened beverages negatively affects fertility and may also reduce the chances of reproductive success with ART [135,136,137].
Red meat	SFAs	Processed red meat has been shown to have a particularly negative effect on fertility [152]. Saturated fat content has been linked to lower semen concentration in males [177] and has a particularly negative effect on ovulation [174]. Eating red meat before IVF had a negative effect on the development of the embryo and the likelihood of clinical pregnancy [178].
Processed foods and fast-food	TFAs	Trans-fatty acids (TFAs) appear to have the most negative impact on fertility. The more TFAs in a woman’s diet, the greater the risk of ovulation disorders [166,168,169,170]. TFAs can also negatively affect semen quality and spermatogenesis [7,154].
Low-fat dairy products	Calcium, vitamins, probiotics	They can support the pro-fertility effect of the diet. It is recommended to choose low-fat dairy products, especially fermented. They have a positive effect on the gut microflora and reduce the risk of type 2 diabetes [7,195,197,198].
Oily sea fish	PUFA, omega-3, fat-soluble vitamins A, D, E, K	Fish are the most pro-fertile sources of fat and protein. They have a positive effect on semen parameters, the course of ovulation, and the success of fertilization [167,177,182,183,184,185,186,187,188,189]. Omega-3 fatty-acid intake should still be recommended to these groups as part of a healthy fertility diet [13].
Nuts, seeds	MUFA, fiber, tocopherols, phytosterols, polyphenols	The consumption of walnuts also has a positive effect on fertility due to the omega-3 content. The use of nuts in the diet may have a beneficial effect on the quality of sperm (longer viability, motility, and morphology) [172,177,185,190,191].
Avocado	MUFA, antioxidants, fiber, folate, potassium	Avocados are an important part of a woman’s fertility diet as they contain much higher amounts of a key nutrient. Associated with better pregnancy outcomes [180].
Plant-based pattern	High in plant protein, antioxidants, fiber, polyphenols, vitamins, minerals. Low-processed food, well-balanced meals. Based on the assumptions of MD.	The plant-based diet, especially high-quality plant foods, can help prevent chronic disease [26]. The plant-based nutritional model can lead to a positive effect on a couple’s fertility [15,16,18,19,20]. Plant-based diets that are high in fiber and polyphenols are also associated with a variety of gut microbiota that produce metabolites that have anti-inflammatory functions that can help manage disease processes, which can prove very beneficial in the context of infertility in couples [131].
Mediterranean pattern	High consumption of plant-derived food, oily sea fish, low-fat dairy and poultry, olive oil, and whole-grain products. Low consumption of simple sugars, red meat, and alcohol.	The MD decreased risk of all-cause mortality, cardiovascular disease, cancer, and other chronic diseases [46,52]. The MD supports proper ovulation in women [9,55,56,57,58,59,60] and has benefits in terms of sperm motility [52]. The MD model increases successful pregnancies by 40% among couples undergoing in vitro fertilization [63]. The MD should be recommended for couples undergoing in vitro fertilization [32].
Western pattern	High-glycemic-index carbohydrates, sweets, beverages, animal protein, SFAs and TFAs, processed foods. Low consumption of FAVs, fiber, vitamins, and sea fish.	It has an antifertility effect and can increase the oxidative stress that causes carbohydrate disturbance. Insulin resistance or diabetes mellitus are related to the deterioration of fertility in women and men, mainly due to the generation of high oxidative stress, which is the main cause leading to an increased risk of infertility and hormonal disorders [16,17,19,86,88].
Low-carb pattern	Low content of carbs, high in fat	There is a lack of reliable research on long-term effects on fertility [141].
Gluten-free pattern	No gluten	Gluten-free diets have been shown to have lower nutritional value compared to traditional diets [144,145]. This is a risky nutritional intervention for fertility, given that it involves eating less fiber and more saturated fatty acids and foods with a higher glycemic index [146].

## Data Availability

Not applicable.

## Abbreviations

MD	Mediterranean diet
GI	Glucose index
BMI	Body mass index
NICE	National Institute for Health and Care Excellence
WHO	World Health Organization
HPG	Hypothalamic–pituitary–gonadal
GnRH	Gonadotropin-releasing hormone
ED	Eating disorders
RED-S	Relative energy deficiency in sport
PCOS	Polycystic ovary syndrome
IVF	In vitro fertilization
NHS II	Nurses Health Cohort Study II
IGF-1	Insulin-like growth factor I
NHS	Nurses’ Health Study
ROS	Reactive oxygen species
OS	Oxidative stress
FAVs	Fruits and vegetables
SOD	Superoxide dismutase
AGEs	Advanced glycation end products
ART	Assisted Reproductive Technology
IS	Isoflavones
TFAs	Trans-fatty acids
PUFA	Polyunsaturated fats
SFAs	Saturated fatty acids
MUFA	Monounsaturated fats
LH	Luteinizing hormone
FSH	Follicle-stimulating hormone

## References

[1] Vander Borght M., Wyns C. (2018). Fertility and Infertility: Definition and Epidemiology. Clin. Biochem..

[2] Gaskins A.J., Chavarro J.E. (2018). Diet and Fertility: A Review. Am. J. Obstet. Gynecol..

[3] Crosignani P.G., Albertini D.F., Anderson R., Bhattacharya S., Evers J.L.H., McLernon D.J., Repping S., Somigliana E., Baird D.T., Diedrich K. (2017). A Prognosis-Based Approach to Infertility: Understanding the Role of Time. Hum. Reprod..

[4] Krueger R.B., Reed G.M., First M.B., Marais A., Kismodi E., Briken P. (2017). Proposals for Paraphilic Disorders in the International Classification of Diseases and Related Health Problems, Eleventh Revision (ICD-11). Arch. Sex. Behav..

[5] Mascarenhas M.N., Flaxman S.R., Boerma T., Vanderpoel S., Stevens G.A. (2012). National, Regional, and Global Trends in Infertility Prevalence Since 1990: A Systematic Analysis of 277 Health Surveys. PLoS Med..

[6] Agarwal A., Mulgund A., Hamada A., Chyatte M.R. (2015). A Unique View on Male Infertility around the Globe. Reprod. Biol. Endocrinol..

[7] Salas-Huetos A., Bulló M., Salas-Salvadó J. (2017). Dietary Patterns, Foods and Nutrients in Male Fertility Parameters and Fecundability: A Systematic Review of Observational Studies. Hum. Reprod. Update.

[8] Vitagliano A., Petre G.C., Francini-Pesenti F., De Toni L., Di Nisio A., Grande G., Foresta C., Garolla A. (2021). Dietary Supplements for Female Infertility: A Critical Review of Their Composition. Nutrients.

[9] Krausz C. (2011). Male Infertility: Pathogenesis and Clinical Diagnosis. Best Pract. Res. Clin. Endocrinol. Metab..

[10] Leaver R.B. (2016). Male Infertility: An Overview of Causes and Treatment Options. Br. J. Nurs..

[11] Skoracka K., Eder P., Łykowska-Szuber L., Dobrowolska A., Krela-Kaźmierczak I. (2020). Diet and Nutritional Factors in Male (In)Fertility—Underestimated Factors. J. Clin. Med..

[12] Carson S.A., Kallen A.N. (2021). Diagnosis and Management of Infertility: A Review. JAMA—J. Am. Med. Assoc..

[13] Panth N., Gavarkovs A., Tamez M., Mattei J. (2018). The Influence of Diet on Fertility and the Implications for Public Health Nutrition in the United States. Front. Public Health.

[14] Simionescu G., Doroftei B., Maftei R., Obreja B.-E., Anton E., Grab D., Ilea C., Anton C. (2021). The Complex Relationship between Infertility and Psychological Distress (Review). Exp. Ther. Med..

[15] Ferramosca A., Zara V. (2022). Diet and Male Fertility: The Impact of Nutrients and Antioxidants on Sperm Energetic Metabolism. Int. J. Mol. Sci..

[16] Skoracka K., Ratajczak A.E., Rychter A.M., Dobrowolska A., Krela-Kaźmierczak I. (2021). Female Fertility and the Nutritional Approach: The Most Essential Aspects. Adv. Nutr..

[17] Arab A., Rafie N., Mansourian M., Miraghajani M., Hajianfar H. (2018). Dietary Patterns and Semen Quality: A Systematic Review and Meta-Analysis of Observational Studies. Andrology.

[18] McGrice M., Porter J. (2017). The Effect of Low Carbohydrate Diets on Fertility Hormones and Outcomes in Overweight and Obese Women: A Systematic Review. Nutrients.

[19] Chavarro J.E., Rich-Edwards J.W., Rosner B.A., Willett W.C. (2009). A Prospective Study of Dietary Carbohydrate Quantity and Quality in Relation to Risk of Ovulatory Infertility. Eur. J. Clin. Nutr..

[20] Chavarro J.E., Rich-Edwards J.W., Rosner B.A., Willett W.C. (2008). Protein Intake and Ovulatory Infertility. Am. J. Obstet. Gynecol..

[21] Marrone G., Guerriero C., Palazzetti D., Lido P., Marolla A., Di Daniele F., Noce A. (2021). Vegan Diet Health Benefits in Metabolic Syndrome. Nutrients.

[22] Aschemann-Witzel J., Gantriis R.F., Fraga P., Perez-Cueto F.J.A. (2021). Plant-Based Food and Protein Trend from a Business Perspective: Markets, Consumers, and the Challenges and Opportunities in the Future. Crit. Rev. Food Sci. Nutr..

[23] Tran E., Dale H.F., Jensen C., Lied G.A. (2020). Effects of Plant-Based Diets on Weight Status: A Systematic Review. Diabetes, Metab. Syndr. Obes. Targets Ther..

[24] Chen Z., Drouin-Chartier J.P., Li Y., Baden M.Y., Manson J.A.E., Willett W.C., Voortman T., Hu F.B., Bhupathiraju S.N. (2021). Changes in Plant-Based Diet Indices and Subsequent Risk of Type 2 Diabetes in Women and Men: Three U.S. Prospective Cohorts. Diabetes Care.

[25] Lee M.F., Eather R., Best T. (2021). Plant-Based Dietary Quality and Depressive Symptoms in Australian Vegans and Vegetarians: A Cross-Sectional Study. BMJ Nutr. Prev. Health.

[26] Millen B.E., Abrams S., Adams-Campbell L., Anderson C.A.M., Brenna J.T., Campbell W.W., Clinton S., Hu F., Nelson M., Neuhouser M.L. (2016). The 2015 Dietary Guidelines Advisory Committee Scientific Report: Development and Major Conclusions. Adv. Nutr..

[27] Silvestris E., de Pergola G., Rosania R., Loverro G. (2018). Obesity as Disruptor of the Female Fertility. Reprod. Biol. Endocrinol..

[28] O’Flynn N. (2014). NICE Fertility: Assessment and Treatment for People with Fertility Problems: NICE guideline. Br. J. Gen. Pract..

[29] WHO Consultation on Obesity (2000). Obesity: Preventing and Managing the Global Epidemic: Report of a WHO Consultation.

[30] Guo D., Xu M., Zhou Q., Wu C., Ju R., Dai J., Arora G. (2019). Is Low Body Mass Index a Risk Factor for Semen Quality? A PRISMA-Compliant Meta-Analysis. Medicine.

[31] Boutari C., Pappas P.D., Mintziori G., Nigdelis M.P., Athanasiadis L., Goulis D.G., Mantzoros C.S. (2020). The Effect of Underweight on Female and Male Reproduction. Metabolism.

[32] Fontana R., Della Torre S. (2016). The Deep Correlation between Energy Metabolism and Reproduction: A View on the Effects of Nutrition for Women Fertility. Nutrients.

[33] Chen W., Pang Y. (2021). Metabolic Syndrome and PCOS: Pathogenesis and the Role of Metabolites. Metabolites.

[34] Nikokavoura E.A., Johnston K.L., Broom J., Wrieden W.L., Rolland C. (2015). Weight Loss for Women with and without Polycystic Ovary Syndrome Following a Very Low-Calorie Diet in a Community-Based Setting with Trained Facilitators for 12 Weeks. Diabetes, Metab. Syndr. Obes. Targets Ther..

[35] Shang Y., Zhou H., He R., Lu W. (2021). Dietary Modification for Reproductive Health in Women With Polycystic Ovary Syndrome: A Systematic Review and Meta-Analysis. Front. Endocrinol..

[36] Kakoly N.S., Earnest A., Teede H.J., Moran L.J., Joham A.E. (2019). The Impact of Obesity on the Incidence of Type 2 Diabetes among Women with Polycystic Ovary Syndrome. Diabetes Care.

[37] Chavarro J.E., Rich-Edwards J.W., Rosner B.A., Willett W.C. (2007). Diet and Lifestyle in the Prevention of Ovulatory Disorder Infertility. Obstet. Gynecol..

[38] Leisegang K., Sengupta P., Agarwal A., Henkel R. (2021). Obesity and Male Infertility: Mechanisms and Management. Andrologia.

[39] Ricci E., Al-Beitawi S., Cipriani S., Alteri A., Chiaffarino F., Candiani M., Gerli S., Viganó P., Parazzini F. (2018). Dietary Habits and Semen Parameters: A Systematic Narrative Review. Andrology.

[40] Chambers T.J., Anderson R.A. (2015). The Impact of Obesity on Male Fertility. Hormones.

[41] El Salam M.A.A. (2018). Obesity, an Enemy of Male Fertility: A Mini Review. Oman Med. J..

[42] Sengupta P., Dutta S., Krajewska-Kulak E. (2017). The Disappearing Sperms: Analysis of Reports Published Between 1980 and 2015. Am. J. Men’s Health.

[43] Salas-Huetos A., James E.R., Aston K.I., Jenkins T.G., Carrell D.T. (2019). Diet and Sperm Quality: Nutrients, Foods and Dietary Patterns. Reprod. Biol..

[44] Giahi L., Mohammadmoradi S., Javidan A., Sadeghi M.R. (2016). Nutritional Modifications in Male Infertility: A Systematic Review Covering 2 Decades. Nutr. Rev..

[45] Schulze M.B., Martínez-González M.A., Fung T.T., Lichtenstein A.H., Forouhi N.G. (2018). Food Based Dietary Patterns and Chronic Disease Prevention. BMJ.

[46] Carlos S., De La Fuente-Arrillaga C., Bes-Rastrollo M., Razquin C., Rico-Campà A., Martínez-González M.A., Ruiz-Canela M. (2018). Mediterranean Diet and Health Outcomes in the SUN Cohort. Nutrients.

[47] Wu S., Zhang X., Zhao X., Hao X., Zhang S., Li P., Tan J. (2022). Preconception Dietary Patterns and Associations With IVF Outcomes: An Ongoing Prospective Cohort Study. Front. Nutr..

[48] Figueroa C., Echeverría G., Villarreal G., Martínez X., Ferreccio C., Rigotti A. (2021). Introducing Plant-Based Mediterranean Diet as a Lifestyle Medicine Approach in Latin America: Opportunities Within the Chilean Context. Front. Nutr..

[49] Hu F.B. (2002). Dietary Pattern Analysis: A New Direction in Nutritional Epidemiology. Curr. Opin. Lipidol..

[50] Khosrorad T., Dolatian M., Riazi H., Mahmoodi Z., Alavimajd H., Shahsavari S., Bakhtiari M. (2015). Comparison of Lifestyle in Fertile and Infertile Couples in Kermanshah during 2013. Iran. J. Reprod. Med..

[51] Gaskins A.J., Nassan F.L., Chiu Y.H., Arvizu M., Williams P.L., Keller M.G., Souter I., Hauser R., Chavarro J.E. (2019). Dietary Patterns and Outcomes of Assisted Reproduction. Am. J. Obstet. Gynecol..

[52] Salas-Huetos A., Babio N., Carrell D.T., Bulló M., Salas-Salvadó J. (2019). Adherence to the Mediterranean Diet Is Positively Associated with Sperm Motility: A Cross-Sectional Analysis. Sci. Rep..

[53] Barrea L., Arnone A., Annunziata G., Muscogiuri G., Laudisio D., Salzano C., Pugliese G., Colao A., Savastano S. (2019). Adherence to the Mediterranean Diet, Dietary Patterns and Body Composition in Women with Polycystic Ovary Syndrome (PCOS). Nutrients.

[54] Griswold M.G., Fullman N., Hawley C., Arian N., Zimsen S.R.M., Tymeson H.D., Venkateswaran V., Tapp A.D., Forouzanfar M.H., Salama J.S. (2018). Alcohol Use and Burden for 195 Countries and Territories, 1990-2016: A Systematic Analysis for the Global Burden of Disease Study 2016. Lancet.

[55] Silvestris E., Lovero D., Palmirotta R. (2019). Nutrition and Female Fertility: An Interdependent Correlation. Front. Endocrinol..

[56] Noli S.A., Ricci E., Cipriani S., Ferrari S., Castiglioni M., La Vecchia I., Somigliana E., Parazzini F. (2020). Dietary Carbohydrate Intake, Dietary Glycemic Load and Outcomes of in Vitro Fertilization: Findings from an Observational Italian Cohort Study. Nutrients.

[57] Koloverou E., Esposito K., Giugliano D., Panagiotakos D. (2014). The Effect of Mediterranean Diet on the Development of Type 2 Diabetes Mellitus: A Meta-Analysis of 10 Prospective Studies and 136,846 Participants. Metabolism.

[58] Abiemo E.E., Alonso A., Nettleton J.A., Steffen L.M., Bertoni A.G., Jain A., Lutsey P.L. (2013). Relationships of the Mediterranean Dietary Pattern with Insulin Resistance and Diabetes Incidence in the Multi-Ethnic Study of Atherosclerosis (MESA). Br. J. Nutr..

[59] Huo R., Du T., Xu Y., Xu W., Chen X., Sun K., Yu X. (2015). Effects of Mediterranean-Style Diet on Glycemic Control, Weight Loss and Cardiovascular Risk Factors among Type 2 Diabetes Individuals: A Meta-Analysis. Eur. J. Clin. Nutr..

[60] Sleiman D., Al-Badri M.R., Azar S.T. (2015). Effect of Mediterranean Diet in Diabetes Control and Cardiovascular Risk Modification: A Systematic Review. Front. Public Health.

[61] Torres-Arce E., Vizmanos B., Babio N., Márquez-Sandoval F., Salas-Huetos A. (2021). Dietary Antioxidants in the Treatment of Male Infertility: Counteracting Oxidative Stress. Biology.

[62] Karayiannis D., Kontogianni M.D., Mendorou C., Douka L., Mastrominas M., Yiannakouris N. (2017). Association between Adherence to the Mediterranean Diet and Semen Quality Parameters in Male Partners of Couples Attempting Fertility. Hum. Reprod..

[63] Vujkovic M., De Vries J.H., Lindemans J., MacKlon N.S., Van Der Spek P.J., Steegers E.A.P., Steegers-Theunissen R.P.M. (2010). The Preconception Mediterranean Dietary Pattern in Couples Undergoing in Vitro Fertilization/Intracytoplasmic Sperm Injection Treatment Increases the Chance of Pregnancy. Fertil. Steril..

[64] Smits R.M., Mackenzie-Proctor R., Yazdani A., Stankiewicz M.T., Jordan V., Showell M.G. (2019). Antioxidants for Male Subfertility. Cochrane Database Syst. Rev..

[65] Christ A., Lauterbach M., Latz E. (2019). Western Diet and the Immune System: An Inflammatory Connection. Immunity.

[66] Yu E., Malik V.S., Hu F.B. (2018). Cardiovascular Disease Prevention by Diet Modification: JACC Health Promotion Series. J. Am. Coll. Cardiol..

[67] Dinu M., Abbate R., Gensini G.F., Casini A., Sofi F. (2017). Vegetarian, Vegan Diets and Multiple Health Outcomes: A Systematic Review with Meta-Analysis of Observational Studies. Crit. Rev. Food Sci. Nutr..

[68] Satija A., Bhupathiraju S.N., Spiegelman D., Chiuve S.E., Manson J.A.E., Willett W., Rexrode K.M., Rimm E.B., Hu F.B. (2017). Healthful and Unhealthful Plant-Based Diets and the Risk of Coronary Heart Disease in U.S. Adults. J. Am. Coll. Cardiol..

[69] McMacken M., Shah S. (2017). A Plant-Based Diet for the Prevention and Treatment of Type 2 Diabetes. J. Geriatr. Cardiol..

[70] Qian F., Liu G., Hu F.B., Bhupathiraju S.N., Sun Q. (2019). Association between Plant-Based Dietary Patterns and Risk of Type 2 Diabetes: A Systematic Review and Meta-Analysis. JAMA Intern. Med..

[71] Austin G., Ferguson J.J.A., Garg M.L. (2021). Effects of Plant-Based Diets on Weight Status in Type 2 Diabetes: A Systematic Review and Meta-Analysis of Randomised Controlled Trials. Nutrients.

[72] Shah B., Newman J.D., Woolf K., Ganguzza L., Guo Y., Allen N., Zhong J., Fisher E.A., Slater J. (2018). Anti-Inflammatory Effects of a Vegan Diet versus the American Heart Association–Recommended Diet in Coronary Artery Disease Trial. J. Am. Heart Assoc..

[73] Menzel J., Biemann R., Longree A., Isermann B., Mai K., Schulze M.B., Abraham K., Weikert C. (2020). Associations of a Vegan Diet with Inflammatory Biomarkers. Sci. Rep..

[74] Medawar E., Huhn S., Villringer A., Veronica Witte A. (2019). The Effects of Plant-Based Diets on the Body and the Brain: A Systematic Review. Transl. Psychiatry.

[75] Kljajic M., Hammadeh M., Wagenpfeil G., Baus S., Sklavounos P., Solomayer E.F., Kasoha M. (2021). Impact of the Vegan Diet on Sperm Quality and Sperm Oxidative Stress Values: A Preliminary Study. J. Hum. Reprod. Sci..

[76] Hall K.D., Guo J., Courville A.B., Boring J., Brychta R., Chen K.Y., Darcey V., Forde C.G., Gharib A.M., Gallagher I. (2021). Effect of a Plant-Based, Low-Fat Diet versus an Animal-Based, Ketogenic Diet on Ad Libitum Energy Intake. Nat. Med..

[77] Pawlak R., Berger J., Hines I. (2018). Iron Status of Vegetarian Adults: A Review of Literature. Am. J. Lifestyle Med..

[78] Adewoyin M., Ibrahim M., Roszaman R., Isa M., Alewi N., Rafa A., Anuar M. (2017). Male Infertility: The Effect of Natural Antioxidants and Phytocompounds on Seminal Oxidative Stress. Diseases.

[79] Özer A., Bakacak M., Kiran H., Ercan Ö., Köstü B., Kanat-Pektaş M., Kilinç M., Aslan F. (2016). Increased Oxidative Stress Is Associated with Insulin Resistance and Infertility in Polycystic Ovary Syndrome. Ginekol. Pol..

[80] Murri M., Luque-ramírez M., Insenser M., Ojeda-ojeda M., Escobar-morreale H.F. (2013). Circulating Markers of Oxidative Stress and Polycystic Ovary Syndrome (Pcos): A Systematic Review and Meta-Analysis. Hum. Reprod. Update.

[81] Agarwal A., Aponte-Mellado A., Premkumar B.J., Shaman A., Gupta S. (2012). The Effects of Oxidative Stress on Female Reproduction: A Review. Reprod. Biol. Endocrinol..

[82] Tremellen K. (2008). Oxidative Stress and Male Infertility—A Clinical Perspective. Hum. Reprod. Update.

[83] Alahmar A. (2019). Role of Oxidative Stress in Male Infertility: An Updated Review. J. Hum. Reprod. Sci..

[84] Wojsiat J., Korczyński J., Borowiecka M., Żbikowska H.M. (2017). The Role of Oxidative Stress in Female Infertility and in Vitro Fertilization. Postepy Hig. Med. Dosw..

[85] Lu J., Wang Z., Cao J., Chen Y., Dong Y. (2018). A Novel and Compact Review on the Role of Oxidative Stress in Female Reproduction. Reprod. Biol. Endocrinol..

[86] Sheweita S., Tilmisany A., Al-Sawaf H. (2005). Mechanisms of Male Infertility: Role of Antioxidants. Curr. Drug Metab..

[87] Sharifi-Rad M., Anil Kumar N.V., Zucca P., Varoni E.M., Dini L., Panzarini E., Rajkovic J., Tsouh Fokou P.V., Azzini E., Peluso I. (2020). Lifestyle, Oxidative Stress, and Antioxidants: Back and Forth in the Pathophysiology of Chronic Diseases. Front. Physiol..

[88] Varani J. (2017). Healthful Eating, the Western Style Diet and Chronic Disease. Approaches Poult. Dairy Vet. Sci..

[89] Chen X.F., Wang L., Wu Y.Z., Song S.Y., Min H.Y., Yang Y., He X., Liang Q., Yi L., Wang Y. (2018). Effect of Puerarin in Promoting Fatty Acid Oxidation by Increasing Mitochondrial Oxidative Capacity and Biogenesis in Skeletal Muscle in Diabetic Rats. Nutr. Diabetes.

[90] Trapp D., Knez W., Sinclair W. (2010). Could a Vegetarian Diet Reduce Exercise-Induced Oxidative Stress? A Review of the Literature. J. Sport. Sci..

[91] Jideani A.I.O., Silungwe H., Takalani T., Omolola A.O., Udeh H.O., Anyasi T.A. (2021). Antioxidant-Rich Natural Fruit and Vegetable Products and Human Health. Int. J. Food Prop..

[92] Carlsen M.H., Halvorsen B.L., Holte K., Bøhn S.K., Dragland S., Sampson L., Willey C., Senoo H., Umezono Y., Sanada C. (2010). The Total Antioxidant Content of More than 3100 Foods, Beverages, Spices, Herbs and Supplements Used Worldwide. Nutr. J..

[93] Salleh N. (2014). Diverse Roles of Prostaglandins in Blastocyst Implantation. Sci. World J..

[94] Joshipura K.J., Ascherio A., Manson J.A.E., Stampfer M.J., Rimm E.B., Speizer F.E., Hennekens C.H., Spiegelman D., Willett W.C. (1999). Fruit and Vegetable Intake in Relation to Risk of Ischemic Stroke. J. Am. Med. Assoc..

[95] Miller V., Mente A., Dehghan M., Rangarajan S., Zhang X., Swaminathan S., Dagenais G., Gupta R., Mohan V., Lear S. (2017). Fruit, Vegetable, and Legume Intake, and Cardiovascular Disease and Deaths in 18 Countries (PURE): A Prospective Cohort Study. Lancet.

[96] Sanderman E.A., Willis S.K., Wise L.A. (2022). Female Dietary Patterns and Outcomes of in Vitro Fertilization (IVF): A Systematic Literature Review. Nutr. J..

[97] Kumar N., Singh A. (2015). Trends of Male Factor Infertility, an Important Cause of Infertility: A Review of Literature. J. Hum. Reprod. Sci..

[98] Buettner G.R. (2011). Superoxide Dismutase in Redox Biology: The Roles of Superoxide and Hydrogen Peroxide. Anticancer Agents Med. Chem..

[99] Wang S., He G., Chen M., Zuo T., Xu W., Liu X. (2017). The Role of Antioxidant Enzymes in the Ovaries. Oxid. Med. Cell. Longev..

[100] Lewandowski Ł., Kepinska M., Milnerowicz H. (2019). The Copper-Zinc Superoxide Dismutase Activity in Selected Diseases. Eur. J. Clin. Investig..

[101] Sabatini L., Wilson C., Lower A., Al-Shawaf T., Grudzinskas J.G. (1999). Superoxide Dismutase Activity in Human Follicular Fluid after Controlled Ovarian Hyperstimulation in Women Undergoing in Vitro Fertilization. Fertil. Steril..

[102] Barrea L., Marzullo P., Muscogiuri G., Di Somma C., Scacchi M., Orio F., Aimaretti G., Colao A., Savastano S. (2018). Source and Amount of Carbohydrate in the Diet and Inflammation in Women with Polycystic Ovary Syndrome. Nutr. Res. Rev..

[103] Insulin Resistance. https://www.ncbi.nlm.nih.gov/books/NBK507839/.

[104] Rama Raju G.A., Jaya Prakash G., Murali Krishna K., Madan K., Siva Narayana T., Ravi Krishna C.H. (2012). Noninsulin-Dependent Diabetes Mellitus: Effects on Sperm Morphological and Functional Characteristics, Nuclear DNA Integrity and Outcome of Assisted Reproductive Technique. Andrologia.

[105] Zhong O., Ji L., Wang J., Lei X., Huang H. (2021). Association of Diabetes and Obesity with Sperm Parameters and Testosterone Levels: A Meta-Analysis. Diabetol. Metab. Syndr..

[106] Lutz W., Leridon H., Aitken R.J., Von Eyben F.E. (2006). Fertility Rates and Future Population Trends: Will Europe’s Birth Rate Recover or Continue to Decline?. Int. J. Androl..

[107] Castela Â., Costa C. (2016). Molecular Mechanisms Associated with Diabetic Endothelial-Erectile Dysfunction. Nat. Rev. Urol..

[108] Lotti F., Corona G., Degli Innocenti S., Filimberti E., Scognamiglio V., Vignozzi L., Forti G., Maggi M. (2013). Seminal, Ultrasound and Psychobiological Parameters Correlate with Metabolic Syndrome in Male Members of Infertile Couples. Andrology.

[109] Martins A.D., Majzoub A., Agawal A. (2019). Metabolic Syndrome and Male Fertility. World J. Men’s Health.

[110] Imani M., Talebi A.R., Fesahat F., Rahiminia T., Seifati S.M., Dehghanpour F. (2021). Sperm Parameters, DNA Integrity, and Protamine Expression in Patients with Type II Diabetes Mellitus. J. Obstet. Gynaecol..

[111] Bhattacharya S.M., Ghosh M., Nandi N. (2014). Diabetes Mellitus and Abnormalities in Semen Analysis. J. Obstet. Gynaecol. Res..

[112] Busetto G.M., Agarwal A., Virmani A., Antonini G., Ragonesi G., Del Giudice F., Micic S., Gentile V., De Berardinis E. (2018). Effect of Metabolic and Antioxidant Supplementation on Sperm Parameters in Oligo-Astheno-Teratozoospermia, with and without Varicocele: A Double-Blind Placebo-Controlled Study. Andrologia.

[113] Braga D.P.D.A.F., Halpern G., Figueira R.D.C.S., Setti A.S., Iaconelli A., Borges E. (2012). Food Intake and Social Habits in Male Patients and Its Relationship to Intracytoplasmic Sperm Injection Outcomes. Fertil. Steril..

[114] Keskes-Ammar L., Feki-Chakroun N., Rebai T., Sahnoun Z., Ghozzi H., Hammami S., Zghal K., Fki H., Damak J., Bahloul A. (2003). Sperm Oxidative Stress and the Effect of an Oral Vitamin E and Selenium Supplement on Semen Quality in Infertile Men. Arch. Androl..

[115] Barratt C.L.R., Björndahl L., De Jonge C.J., Lamb D.J., Martini F.O., McLachlan R., Oates R.D., van der Poel S., John B.S., Sigman M. (2017). The Diagnosis of Male Infertility: An Analysis of the Evidence to Support the Development of Global WHO Guidance-Challenges and Future Research Opportunities. Hum. Reprod. Update.

[116] Cardoso J.P., Cocuzza M., Elterman D. (2019). Optimizing Male Fertility: Oxidative Stress and the Use of Antioxidants. World J. Urol..

[117] Kothari R.P., Chaudhari A.R. (2016). Zinc Levels in Seminal Fluid in Infertile Males and Its Relation with Serum Free Testosterone. J. Clin. Diagn. Res..

[118] Yamaguchi S., Miura C., Kikuchi K., Celino F.T., Agusa T., Tanabe S., Miura T. (2009). Zinc Is an Essential Trace Element for Spermatogenesis. Proc. Natl. Acad. Sci. USA.

[119] Mirnamniha M., Faroughi F., Tahmasbpour E., Ebrahimi P., Harchegani A.B. (2019). An Overview on Role of Some Trace Elements in Human Reproductive Health, Sperm Function and Fertilization Process. Rev. Environ. Health.

[120] Fallah A., Mohammad-Hasani A., Colagar A.H. (2018). Zinc Is an Essential Element for Male Fertility: A Review of Zn Roles in Men’s Health, Germination, Sperm Quality, and Fertilization. J. Reprod. Infertil..

[121] Gammoh N.Z., Rink L. (2017). Zinc in Infection and Inflammation. Nutrients.

[122] Schisterman E.F., Sjaarda L.A., Clemons T., Carrell D.T., Perkins N.J., Johnstone E., Lamb D., Chaney K., Van Voorhis B.J., Ryan G. (2020). Effect of Folic Acid and Zinc Supplementation in Men on Semen Quality and Live Birth among Couples Undergoing Infertility Treatment: A Randomized Clinical Trial. JAMA—J. Am. Med. Assoc..

[123] Lerda D. (1992). Study of Sperm Characteristics in Persons Occupationally Exposed to Lead. Am. J. Ind. Med..

[124] Hawkes W.C., Turek P.J. (2001). Effects of Dietary Selenium on Sperm Motility in Healthy Men. J. Androl..

[125] Mintziori G., Mousiolis A., Duntas L.H., Goulis D.G. (2020). Evidence for a Manifold Role of Selenium in Infertility. Hormones.

[126] Majzoub A., Agarwal A. (2018). Systematic Review of Antioxidant Types and Doses in Male Infertility: Benefits on Semen Parameters, Advanced Sperm Function, Assisted Reproduction and Live-Birth Rate. Arab J. Urol..

[127] Durairajanayagam D., Agarwal A., Ong C., Prashast P. (2014). Lycopene and Male Infertility. Asian J. Androl..

[128] Bakaloudi D.R., Halloran A., Rippin H.L., Oikonomidou A.C., Dardavesis T.I., Williams J., Wickramasinghe K., Breda J., Chourdakis M. (2021). Intake and Adequacy of the Vegan Diet. A Systematic Review of the Evidence. Clin. Nutr..

[129] Sakkas H., Bozidis P., Touzios C., Kolios D., Athanasiou G., Athanasopoulou E., Gerou I., Gartzonika C. (2020). Nutritional Status and the Influence of the Vegan Diet on the Gut Microbiota and Human Health. Medicina.

[130] Neufingerl N., Eilander A. (2022). Nutrient Intake and Status in Adults Consuming Plant-Based Diets Compared to Meat-Eaters: A Systematic Review. Nutrients.

[131] Craig W.J., Mangels A.R., Fresán U., Marsh K., Miles F.L., Saunders A.V., Haddad E.H., Heskey C.E., Johnston P., Larson-meyer E. (2021). The Safe and Effective Use of Plant-based Diets with Guidelines for Health Professionals. Nutrients.

[132] Toledo E., Lopez-Del Burgo C., Ruiz-Zambrana A., Donazar M., Navarro-Blasco Í., Martínez-González M.A., De Irala J. (2011). Dietary Patterns and Difficulty Conceiving: A Nested Case-Control Study. Fertil. Steril..

[133] Willis S.K., Wise L.A., Wesselink A.K., Rothman K.J., Mikkelsen E.M., Tucker K.L., Trolle E., Hatch E.E. (2020). Glycemic Load, Dietary Fiber, and Added Sugar and Fecundability in 2 Preconception Cohorts. Am. J. Clin. Nutr..

[134] Walczak-Jedrzejowska R., Wolski J.K., Slowikowska-Hilczer J. (2013). The Role of Oxidative Stress and Antioxidants in Male Fertility. Cent. Eur. J. Urol..

[135] Hatch E.E., Wesselink A.K., Hahn K.A., Michiel J.J., Mikkelsen E.M., Sorensen H.T., Rothman K.J., Wise L.A. (2018). Intake of Sugar-Sweetened Beverages and Fecundability in a North American Preconception Cohort. Epidemiology.

[136] Schliep K.C., Schisterman E.F., Mumford S.L., Pollack A.Z., Perkins N.J., Ye A., Zhang C.J., Stanford J.B., Porucznik C.A., Hammoud A.O. (2013). Energy-Containing Beverages: Reproductive Hormones and Ovarian Function in the Biocycle Study1-3. Am. J. Clin. Nutr..

[137] Lim S.X., Loy S.L., Colega M.T., Lai J.S., Godfrey K.M., Lee Y.S., Tan K.H., Yap F., Shek L.P.C., Chong Y.S. (2022). Prepregnancy Adherence to Plant-Based Diet Indices and Exploratory Dietary Patterns in Relation to Fecundability. Am. J. Clin. Nutr..

[138] Chiu Y.H., Afeiche M.C., Gaskins A.J., Williams P.L., Mendiola J., Jorgensen N., Swan S.H., Chavarro J.E. (2014). Sugar-Sweetened Beverage Intake in Relation to Semen Quality and Reproductive Hormone Levels in Young Men. Hum. Reprod..

[139] Hatch E.E., Wise L.A., Mikkelsen E.M., Christensen T., Riis A.H., Sørensen H.T., Rothman K.J. (2012). Caffeinated Beverage and Soda Consumption and Time to Pregnancy. Epidemiology.

[140] Kazemi M., Hadi A., Pierson R.A., Lujan M.E., Zello G.A., Chilibeck P.D. (2021). Effects of Dietary Glycemic Index and Glycemic Load on Cardiometabolic and Reproductive Profiles in Women with Polycystic Ovary Syndrome: A Systematic Review and Meta-Analysis of Randomized Controlled Trials. Adv. Nutr..

[141] Caprio M., Infante M., Moriconi E., Armani A., Fabbri A., Mantovani G., Mariani S., Lubrano C., Poggiogalle E., Migliaccio S. (2019). Very-Low-Calorie Ketogenic Diet (VLCKD) in the Management of Metabolic Diseases: Systematic Review and Consensus Statement from the Italian Society of Endocrinology (SIE). J. Endocrinol. Investig..

[142] Gaskins A.J., Mumford S.L., Zhang C., Wactawski-Wende J., Hovey K.M., Whitcomb B.W., Howards P.P., Perkins N.J., Yeung E., Schisterman E.F. (2009). Effect of Daily Fiber Intake on Reproductive Function: The BioCycle Study. Am. J. Clin. Nutr..

[143] Colombo O., Pinelli G., Comelli M., Marchetti P., Sieri S., Brighenti F., Nappi R.E., Tagliabue A. (2009). Dietary Intakes in Infertile Women a Pilot Study. Nutr. J..

[144] Rostami K., Bold J., Parr A., Johnson M.W. (2017). Gluten-Free Diet Indications, Safety, Quality, Labels, and Challenges. Nutrients.

[145] Vici G., Belli L., Biondi M., Polzonetti V. (2016). Gluten Free Diet and Nutrient Deficiencies: A Review. Clin. Nutr..

[146] Diez-Sampedro A., Olenick M., Maltseva T., Flowers M. (2019). A Gluten-Free Diet, Not an Appropriate Choice without a Medical Diagnosis. J. Nutr. Metab..

[147] Kemp B., Grooten H.J.G., Den Hartog L.A., Luiting P., Verstegen M.W.A. (1988). The Effect of a High Protein Intake on Sperm Production in Boars at Two Semen Collection Frequencies. Anim. Reprod. Sci..

[148] Farshchi H., Rane A., Love A., Kennedy R.L. (2007). Diet and Nutrition in Polycystic Ovary Syndrome (PCOS): Pointers for Nutritional Management. J. Obstet. Gynaecol..

[149] Mumford S.L., Alohali A., Wactawski-Wende J. (2015). Dietary Protein Intake and Reproductive Hormones and Ovulation: The BioCycle Study. Fertil. Steril..

[150] Walters K.A., Handelsman D.J. (2018). Role of Androgens in the Ovary. Mol. Cell. Endocrinol..

[151] Souter I., Chiu Y.H., Batsis M., Afeiche M.C., Williams P.L., Hauser R., Chavarro J.E. (2017). The Association of Protein Intake (Amount and Type) with Ovarian Antral Follicle Counts among Infertile Women: Results from the EARTH Prospective Study Cohort. BJOG Int. J. Obstet. Gynaecol..

[152] Zhang B., Zhang B., Zhang B., Zhang B., Zhou W., Zhou W., Zhou W., Zhou W., Shi Y., Shi Y. (2020). Lifestyle and Environmental Contributions to Ovulatory Dysfunction in Women of Polycystic Ovary Syndrome. BMC Endocr. Disord..

[153] Mendiola J., Torres-Cantero A.M., Moreno-Grau J.M., Ten J., Roca M., Moreno-Grau S., Bernabeu R. (2009). Food Intake and Its Relationship with Semen Quality: A Case-Control Study. Fertil. Steril..

[154] Chavarro J.E., Furtado J., Toth T.L., Ford J., Keller M., Campos H., Hauser R. (2011). Trans-Fatty Acid Levels in Sperm Are Associated with Sperm Concentration among Men from an Infertility Clinic. Fertil. Steril..

[155] Xia W., Chiu Y.H., Williams P.L., Gaskins A.J., Toth T.L., Tanrikut C., Hauser R., Chavarro J.E. (2015). Men’s Meat Intake and Treatment Outcomes among Couples Undergoing Assisted Reproduction. Fertil. Steril..

[156] Nassan F.L., Chiu Y.H., Vanegas J.C., Gaskins A.J., Williams P.L., Ford J.B., Attaman J., Hauser R., Chavarro J.E. (2018). Intake of Protein-Rich Foods in Relation to Outcomes of Infertility Treatment with Assisted Reproductive Technologies. Am. J. Clin. Nutr..

[157] Unfer V., Casini M.L., Gerli S., Costabile L., Mignosa M., Di Renzo G.C. (2004). Phytoestrogens May Improve the Pregnancy Rate in in Vitro Fertilization-Embryo Transfer Cycles: A Prospective, Controlled, Randomized Trial. Fertil. Steril..

[158] Mumford S.L., Sundaram R., Schisterman E.F., Sweeney A.M., Barr D.B., Rybak M.E., Maisog J.M., Parker D.L., Pfeiffer C.M., Louis G.M.B. (2014). Higher Urinary Lignan Concentrations in Women but Not Men Are Positively Associated with Shorter Time to Pregnancy. J. Nutr..

[159] Vanegas J.C., Afeiche M.C., Gaskins A.J., Mínguez-Alarcón L., Williams P.L., Wright D.L., Toth T.L., Hauser R., Chavarro J.E. (2015). Soy Food Intake and Treatment Outcomes of Women Undergoing Assisted Reproductive Technology. Fertil. Steril..

[160] Rizzo G., Feraco A., Storz M.A., Lombardo M. (2022). The Role of Soy and Soy Isoflavones on Women’s Fertility and Related Outcomes: An Update. J. Nutr. Sci..

[161] Hamilton-Reeves J.M., Vazquez G., Duval S.J., Phipps W.R., Kurzer M.S., Messina M.J. (2010). Clinical Studies Show No Effects of Soy Protein or Isoflavones on Reproductive Hormones in Men: Results of a Meta-Analysis. Fertil. Steril..

[162] Van Die M.D., Bone K.M., Williams S.G., Pirotta M.V. (2014). Soy and Soy Isoflavones in Prostate Cancer: A Systematic Review and Meta-Analysis of Randomized Controlled Trials. BJU Int..

[163] Reed K.E., Camargo J., Hamilton-Reeves J., Kurzer M., Messina M. (2021). Neither Soy nor Isoflavone Intake Affects Male Reproductive Hormones: An Expanded and Updated Meta-Analysis of Clinical Studies. Reprod. Toxicol..

[164] Chavarro J.E., Toth T.L., Sadio S.M., Hauser R. (2008). Soy Food and Isoflavone Intake in Relation to Semen Quality Parameters among Men from an Infertility Clinic. Hum. Reprod..

[165] Jacobsen B.K., Jaceldo-Siegl K., Knutsen S.F., Fan J., Oda K., Fraser G.E. (2014). Soy Isoflavone Intake and the Likelihood of Ever Becoming a Mother: The Adventist Health Study-2. Int. J. Women’s. Health.

[166] Chavarro J.E., Rich-Edwards J.W., Rosner B.A., Willett W.C. (2007). Dietary Fatty Acid Intakes and the Risk of Ovulatory Infertility. Am. J. Clin. Nutr..

[167] Bauer J.L., Kuhn K., Bradford A.P., Al-Safi Z.A., Harris M.A., Eckel R.H., Robledo C.Y., Malkhasyan A., Johnson J., Gee N.R. (2019). Reduction in FSH Throughout the Menstrual Cycle After Omega-3 Fatty Acid Supplementation in Young Normal Weight but Not Obese Women. Reprod. Sci..

[168] Lefevre M., Lovejoy J.C., Smith S.R., Delany J.P., Champagne C., Most M.M., Denkins Y., De Jonge L., Rood J., Bray G.A. (2005). Comparison of the Acute Response to Meals Enriched with Cis- or Trans-Fatty Acids on Glucose and Lipids in Overweight Individuals with Differing FABP2 Genotypes. Metabolism.

[169] Belani M., Purohit N., Pillai P., Gupta S., Gupta S. (2014). Modulation of Steroidogenic Pathway in Rat Granulosa Cells with Subclinical Cd Exposure and Insulin Resistance: An Impact on Female Fertility. Biomed Res. Int..

[170] Baer D.J., Judd J.T., Clevidence B.A., Tracy R.P. (2004). Dietary Fatty Acids Affect Plasma Markers of Inflammation in Healthy Men Fed Controlled Diets: A Randomized Crossover Study. Am. J. Clin. Nutr..

[171] Diamanti-Kandarakis E., Dunaif A. (2012). Insulin Resistance and the Polycystic Ovary Syndrome Revisited: An Update on Mechanisms and Implications. Endocr. Rev..

[172] Wise L.A., Wesselink A.K., Tucker K.L., Saklani S., Mikkelsen E.M., Cueto H., Riis A.H., Trolle E., McKinnon C.J., Hahn K.A. (2018). Dietary Fat Intake and Fecundability in 2 Preconception Cohort Studies. Am. J. Epidemiol..

[173] de Souza R.J., Mente A., Maroleanu A., Cozma A.I., Ha V., Kishibe T., Uleryk E., Budylowski P., Schünemann H., Beyene J. (2015). Intake of Saturated and Trans Unsaturated Fatty Acids and Risk of All Cause Mortality, Cardiovascular Disease, and Type 2 Diabetes: Systematic Review and Meta-Analysis of Observational Studies. BMJ.

[174] Çekici H., Akdevelioğlu Y. (2019). The Association between Trans Fatty Acids, Infertility and Fetal Life: A Review. Hum. Fertil..

[175] Douglas C.C., Norris L.E., Oster R.A., Darnell B.E., Azziz R., Gower B.A. (2006). Difference in Dietary Intake between Women with Polycystic Ovary Syndrome and Healthy Controls. Fertil. Steril..

[176] Carmina E., Legro R.S., Stamets K., Lowell J., Lobo R.A. (2003). Difference in Body Weight between American and Italian Women with Polycystic Ovary Syndrome: Influence of the Diet. Hum. Reprod..

[177] Attaman J.A., Toth T.L., Furtado J., Campos H., Hauser R., Chavarro J.E. (2012). Dietary Fat and Semen Quality among Men Attending a Fertility Clinic. Hum. Reprod..

[178] Braga D.P.A.F., Halpern G., Setti A.S., Figueira R.C.S., Iaconelli A., Borges E. (2015). The Impact of Food Intake and Social Habits on Embryo Quality and the Likelihood of Blastocyst Formation. Reprod. Biomed. Online.

[179] Axmon A., Rylander L., Strömberg U., Hagmar L. (2002). Female Fertility in Relation to the Consumption of Fish Contaminated with Persistent Organochlorine Compounds. Scand. J. Work. Environ. Health.

[180] Comerford K.B., Ayoob K.T., Murray R.D., Atkinson S.A. (2016). The Role of Avocados in Maternal Diets during the Periconceptional Period, Pregnancy, and Lactation. Nutrients.

[181] Mumford S.L., Browne R.W., Kim K., Nichols C., Wilcox B., Silver R.M., Connell M.T., Holland T.L., Kuhr D.L., Omosigho U.R. (2018). Preconception Plasma Phospholipid Fatty Acids and Fecundability. J. Clin. Endocrinol. Metab..

[182] Falsig A.M.L., Gleerup C.S., Knudsen U.B. (2019). The Influence of Omega-3 Fatty Acids on Semen Quality Markers: A Systematic PRISMA Review. Andrology.

[183] Moran L.J., Tsagareli V., Noakes M., Norman R. (2016). Altered Preconception Fatty Acid Intake Is Associated with Improved Pregnancy Rates in Overweight and Obesewomen Undertaking in Vitro Fertilisation. Nutrients.

[184] Salas-Huetos A., Arvizu M., Mínguez-Alarcón L., Mitsunami M., Ribas-Maynou J., Yeste M., Ford J.B., Souter I., Chavarro J.E. (2022). Women’s and Men’s Intake of Omega-3 Fatty Acids and Their Food Sources and Assisted Reproductive Technology Outcomes. Am. J. Obstet. Gynecol..

[185] Mumford S.L., Chavarro J.E., Zhang C., Perkins N.J., Sjaarda L.A., Pollack A.Z., Schliep K.C., Michels K.A., Zarek S.M., Plowden T.C. (2016). Dietary Fat Intake and Reproductive Hormone Concentrations and Ovulation in Regularly Menstruating Women. Am. J. Clin. Nutr..

[186] Nehra D., Le H.D., Fallon E.M., Carlson S.J., Woods D., White Y.A., Pan A.H., Guo L., Rodig S.J., Tilly J.L. (2012). Prolonging the Female Reproductive Lifespan and Improving Egg Quality with Dietary Omega-3 Fatty Acids. Aging Cell.

[187] Hammiche F., Vujkovic M., Wijburg W., De Vries J.H.M., MacKlon N.S., Laven J.S.E., Steegers-Theunissen R.P.M. (2011). Increased Preconception Omega-3 Polyunsaturated Fatty Acid Intake Improves Embryo Morphology. Fertil. Steril..

[188] Wathes D.C., Abayasekara D.R.E., Aitken R.J. (2007). Polyunsaturated Fatty Acids in Male and Female Reproduction. Biol. Reprod..

[189] Ruan Y.C., Guo J.H., Liu X., Zhang R., Tsang L.L., Da Dong J., Chen H., Yu M.K., Jiang X., Zhang X.H. (2012). Activation of the Epithelial Na+ Channel Triggers Prostaglandin E2 Release and Production Required for Embryo Implantation. Nat. Med..

[190] Salas-Huetos A., Moraleda R., Giardina S., Anton E., Blanco J., Salas-Salvadó J., Bulló M. (2018). Effect of Nut Consumption on Semen Quality and Functionality in Healthy Men Consuming a Western-Style Diet: A Randomized Controlled Trial. Am. J. Clin. Nutr..

[191] Robbins W.A., Xun L., FitzGerald L.Z., Esguerra S., Henning S.M., Carpenter C.L. (2012). Walnuts Improve Semen Quality in Men Consuming a Western-Style Diet: Randomized Control Dietary Intervention Trial. Biol. Reprod..

[192] Afeiche M.C., Chiu Y.H., Gaskins A.J., Williams P.L., Souter I., Wright D.L., Hauser R., Chavarro J.E. (2016). Dairy Intake in Relation to in Vitro Fertilization Outcomes among Women from a Fertility Clinic. Hum. Reprod..

[193] Kim K., Wactawski-Wende J., Michels K.A., Plowden T.C., Chaljub E.N., Sjaarda L.A., Mumford S.L. (2017). Dairy Food Intake Is Associated with Reproductive Hormones and Sporadic Anovulation among Healthy Premenopausal Women. J. Nutr..

[194] Chavarro J.E., Rich-Edwards J.W., Rosner B., Willett W.C. (2007). A Prospective Study of Dairy Foods Intake and Anovulatory Infertility. Hum. Reprod..

[195] Greenlee A.R., Arbuckle T.E., Chyou P.H. (2003). Risk Factors for Female Infertility in an Agricultural Region. Epidemiology.

[196] Salas-Salvadó J., Guasch-Ferré M., Díaz-López A., Babio N. (2017). Yogurt and Diabetes: Overview of Recent Observational Studies. J. Nutr..

[197] Wen L., Duffy A. (2017). Factors Influencing the Gut Microbiota, Inflammation, and Type 2 Diabetes. J. Nutr..

[198] Janiszewska J., Ostrowska J., Szostak-Węgierek D. (2020). Milk and Dairy Products and Their Impact on Carbohydrate Metabolism and Fertility—A Potential Role in the Diet of Women with Polycystic Ovary Syndrome. Nutrients.

[199] Afeiche M.C., Bridges N.D., Williams P.L., Gaskins A.J., Tanrikut C., Petrozza J.C., Hauser R., Chavarro J.E. (2014). Dairy Intake and Semen Quality among Men Attending a Fertility Clinic. Fertil. Steril..

